# Enhancement of host defense against *Helicobacter pylori* infection through modulation of the gastrointestinal microenvironment by *Lactiplantibacillus plantarum* Lp05

**DOI:** 10.3389/fimmu.2024.1469885

**Published:** 2025-01-17

**Authors:** Yao Dong, Mei Han, Yongmei Qi, Ying Wu, Zhipeng Zhou, Dacheng Jiang, Zhonghui Gai

**Affiliations:** ^1^ Department of Research and Development, Wecare Probiotics Co., Ltd., Suzhou, China; ^2^ Department of Food Quality and Safety, Shanghai Business School, Shanghai, China; ^3^ College of Food and Bioengineering, Henan University of Science and Technology, Luoyang, China; ^4^ Food Science and Nutrition, University of Leeds, Leeds, United Kingdom

**Keywords:** *Helicobacter pylori*, *Lactiplantibacillus plantarum*, gut microbiota, gastric microenvironment, inflammatory markers

## Abstract

**Objective:**

This study aimed to assess the impact of *Lactiplantibacillus plantarum* Lp05 (Lp05) on the gastrointestinal microbiome and pathophysiological status of mice infected with *Helicobacter pylori* (*H. pylori*), exploring its potential as a probiotic treatment for *H. pylori* infections.

**Methods:**

*In vitro*, the interaction between Lp05 and *H. pylori* was analyzed using laser confocal and scanning electron microscopy. *In vivo*, C57BL/6 mice infected with *H. pylori* were treated with Lp05 and divided into six groups: control, model, quadruple therapy, and three dosage levels of Lp05 (2×10^7^, 2×10^8^, 2×10^9^ CFU/mouse/day). Over six weeks, the impact of Lp05 on the gastrointestinal microbiome and physiological markers was assessed. Measurements included digestive enzymes (α-amylase, pepsin, cellulase), inflammatory markers (interleukin-17A, interleukin-23, interleukin-10, interferon-β, interferon-γ, FoxP3, endothelin, IP-10, TGF-β1), oxidative stress markers (catalase, malondialdehyde, superoxide dismutase, myeloperoxidase), and tissue pathology (via modified Warthin-Starry silver and H&E staining). Microbial community structure in the stomach and intestines was evaluated through 16S rRNA gene sequencing.

**Results:**

*In vitro* studies showed Lp05 and *H. pylori* formed co-aggregates, with Lp05 potentially disrupting *H. pylori* cell structure, reducing its stomach colonization. *In vivo*, Lp05 significantly lowered gastric mucosal urease activity and serum *H. pylori*-IgG antibody levels in infected mice (*p* < 0.01). It also mitigated pathological changes in the stomach and duodenum, decreased inflammatory responses (ET, IL-17A, IL-23, TGF-beta1, and IP-10, *p* < 0.01 for all), and enhanced antioxidant enzyme activities (CAT and SOD, *p* < 0.01) while reducing MDA and MPO levels (*p* < 0.01), combating oxidative stress from *H. pylori* infection. Lp05 treatment significantly modified the intestinal and gastric microbiota, increasing beneficial bacteria like *Lactobacillus* and *Ligilactobacillus*, and decreasing harmful bacteria such as *Olsenella*, linked to pathological conditions.

**Conclusion:**

Lp05 effectively modulates the gastrointestinal microbiome, reduces inflammation and oxidative stress, and suppresses *H. pylori*, promising for probiotic therapies with further research needed to refine its clinical use.

## Graphical Abstract

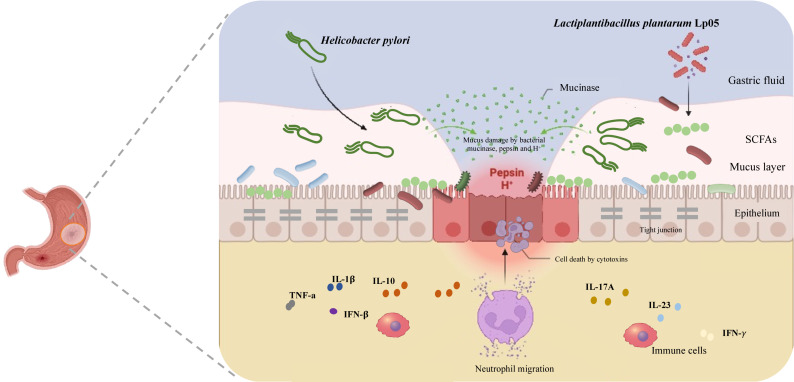

## Introduction

Medically, *Helicobacter pylori* (*H. pylori*) is a microaerophilic, spiral-shaped, Gram-negative bacterium that commonly inhabits the human stomach (1). Discovered in 1982, it profoundly influences gastroenterology and infectious diseases by forming persistent, often lifelong infections and adapting to acidic gastric conditions (2). *H. pylori* adheres to gastric mucosa, forms biofilms, and employs strategies like motility, adhesion, urease activity, and cytotoxin production for its survival and pathogenicity (3, 4). By breaking down urea into ammonia, it neutralizes stomach acidity, maintaining a conducive environment and a stable nitrogen source for itself (5). Additionally, *H. pylori* triggers chronic inflammation through immune activation, which aids its survival and may lead to gastric damage and cancer (6). It modifies the protective mucosal layer to enhance colonization and evade immune responses. Though often asymptomatic, *H. pylori* infection correlates with significant gastrointestinal diseases such as peptic ulcers, non-ulcer dyspepsia, and gastric cancer (6, 7), the latter being strongly associated with its classification by the International Agency for Research on Cancer (IARC) as a Group 1 carcinogen (8). Addressing *H. pylori* infection is essential, particularly in regions with high gastric cancer rates, prompting ongoing research to better understand its pathogenic mechanisms, improve diagnostics, and develop more effective treatments to reduce its health impact.

The standard triple therapy for *H. pylori* infection, endorsed by the Maastricht IV/Florence Consensus, comprises two antibiotics (usually amoxicillin and clarithromycin) and a proton pump inhibitor (PPI) (9). This regimen faces hurdles like antibiotic resistance, adverse effects, and high costs (10). When clarithromycin resistance occurs, a bismuth-containing quadruple therapy (BQT) is often substituted (11). Although PPIs are crucial for enhancing treatment efficacy and inhibiting bacterial growth, they may increase risks of intestinal infections and acute kidney injury (10). With rising global antibiotic resistance, especially to clarithromycin and levofloxacin, treatment success rates are under strain. The latest Maastricht VI/Florence Consensus advises using triple or bismuth quadruple therapy in areas with low resistance (12). Nevertheless, the broad use of antibiotics has prompted the exploration of alternative treatments (13). Given these circumstances, probiotics have gained significant interest for their safety, immunomodulatory effects, and antimicrobial properties (14). These live microorganisms benefit the host by altering the microbial composition at specific sites (15). Lactobacilli, thriving in the stomach’s acidic conditions (pH 4-6), are particularly suited for treating *H. pylori* infections (4, 16, 17). Research indicates that certain probiotics can directly inhibit *H. pylori* growth or modify its lifecycle by enhancing host immune responses, offering a non-antibiotic strategy for managing these infections (4, 16, 17). For example, *Lactobacillus johnsonii* No. 1088 has shown significant antimicrobial effects in *H. pylori*-infected mice (18). Moreover, combining probiotics with traditional BQT significantly boosts eradication rates, achieving a 92% cure rate compared to 86.8% without probiotics (19). These insights underline the critical role of gut microbiota in disease progression and support the development of innovative treatment approaches.

While *Lactiplantibacillus plantarum* is well-studied as a probiotic, its specific anti-*H. pylori* mechanisms and effects on host microbial communities are not fully understood, especially how it modulates microbial communities and immune responses to diminish *H. pylori* stomach colonization. *In vitro*, *L. plantarum* Lp05 (Lp05) has demonstrated the ability to inhibit *H. pylori* growth, highlighting its antimicrobial potential. This study focuses on Lp05, analyzing its impact on gastric mucosal urease activity, immune responses, and gastrointestinal microbiota in an *H. pylori*-infected mouse model. The goal is to explore the mechanisms of its inhibitory effects on *H. pylori* and assess its potential as a novel treatment approach, providing a foundation for future therapeutic strategies.

## Methods and materials

### Preparation of strains

Strain Lp05 was provided by Wecare Probiotics Co., Ltd. (Suzhou, China). The strain was cultured in De Man, Rogosa, and Sharpe (MRS) medium at 37°C for 18 h (20). Bacterial cells were harvested by centrifugation at 6000 *× g* for 8 min and resuspended in sterile water to achieve a final concentration of 1×10^9^ CFU/mL.

*H. pylori* SS1, obtained from BeNa Culture Collection, was revived from -80°C storage on Columbia agar plates supplemented with 5% sterile defibrinated sheep blood. After being revived for two generations, it was cultured on Columbia blood agar plates for 48 h, washed with BHI broth, and the bacterial suspension adjusted to 1×10^10^ CFU/mL (21).

### Confocal laser scanning microscopy

Cultured *H. pylori* SS1 was centrifuged and washed twice with artificial gastric juice (22), then resuspended to adjust the OD_600_ to approximately 0.50. After staining with CFDA SE dye and incubating at 37°C for 30 min, excess dye was removed by centrifuging the bacteria in PBS. The pre-stained *H. pylori* SS1 was then mixed with Lp05 in equal volumes, vortexed for at least 10 s, and incubated at 37°C for 2 h. A sample of this mixture was placed on a slide and examined under a confocal laser scanning microscope (E_x_ = 494 nm, E_m_ = 521 nm) (23).

### Scanning electron microscopy

Strain Lp05 was centrifuged, washed twice with PBS, and resuspended in sterile PBS. Similarly, *H. pylori* SS1 was washed and resuspended in artificial gastric juice. Both suspensions were adjusted to an OD_600_ of approximately 0.50 and mixed equally to induce co-aggregation. After 2 h at 25°C, the aggregates were fixed overnight in 2.5% glutaraldehyde at 4°C, dehydrated in a graded ethanol series, and gold-sputter coated. The samples were then scanned under a scanning electron microscope (24).

### Establishment of *H. pylori* infection model in mice

**Figure 1:**
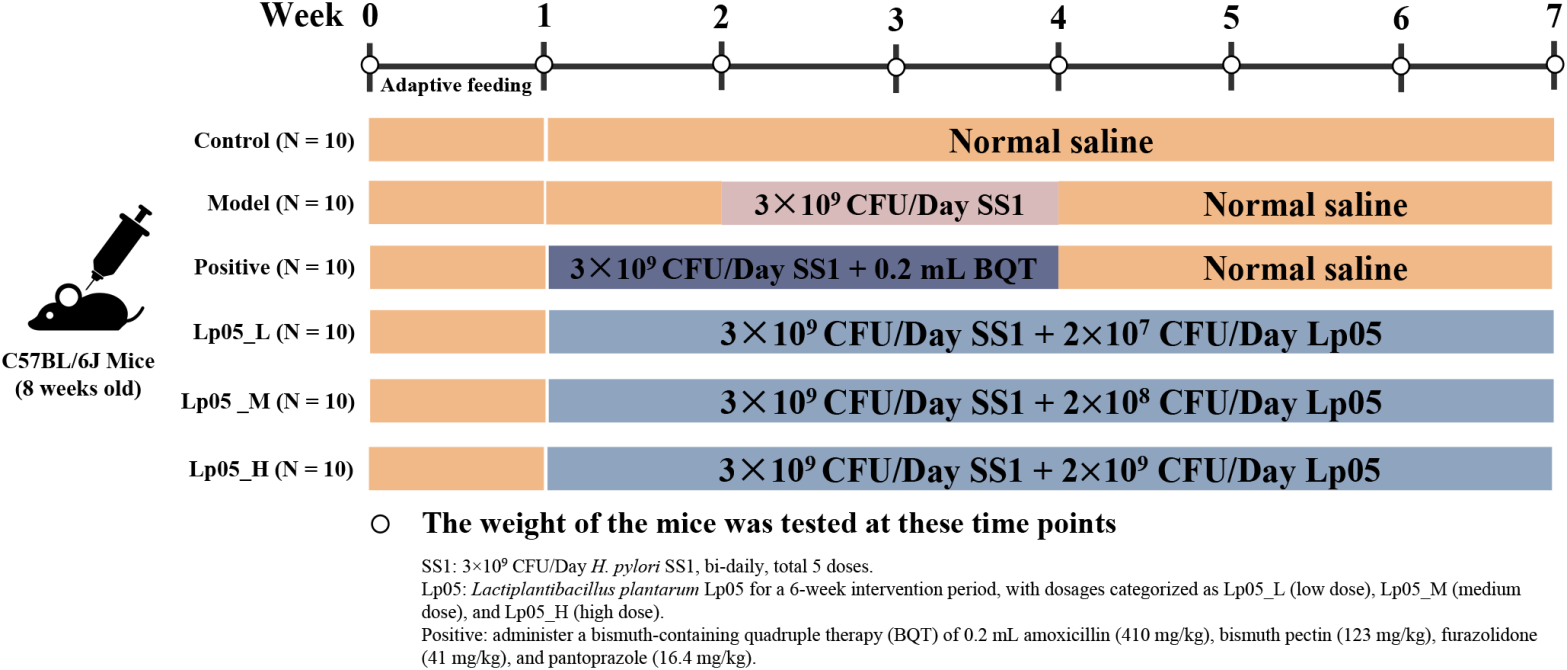
Experimental design diagram of the *Helicobacter pylori* SS1 infection model in mice.

All procedures involving mice were approved by the Experimental Animal Ethics Committee of Zhengzhou University (License No: SYXK(Yu)2021-0011) and followed the ARRIVE guidelines 2.0 (25). To establish the *H. pylori* infection model, 60 eight-week-old female SPF-grade C57BL/6 mice (SPF Biotechnology Co., Ltd, Beijing, China) were acclimatized for a week and then divided into six groups of ten each for a six-week experiment (26) (**Figure 1**). The control (CTL) group received daily gavage of 0.2 mL saline throughout the experiment. From the second week, the five treatment groups started modeling *H. pylori* SS1 infection with bi-daily gavages of a 0.2 mL 0.9% NaCl suspension containing 1×10^10^ CFU/mL *H. pylori* for a total of five times. The model control group (MC) followed the same saline regimen and only *H. pylori*. The quadruple therapy group (PG) received a normal diet in the first week and post-infection, daily gavages of a mix containing amoxicillin (410 mg/kg), bismuth pectin (123 mg/kg), furazolidone (41 mg/kg), and pantoprazole (16.4 mg/kg) during the second and third weeks, followed by 0.2 mL 0.9% NaCl until week six. The Lp05 low, medium, and high dose groups (Lp05_L, Lp05_M, Lp05_H) received daily gavages of 0.2 mL with Lp05 at 1×10^8^, 1×10^9^, and 1×10^10^ CFU/mL respectively, from the first week and began *H. pylori* infection the same as MC group from the second week. All mice were kept at 25 ± 2°C with bedding changed two to three times weekly and weighed regularly.

### Processing of mice blood and tissue samples

After the final gavage, mice fasted for 12 hours were anesthetized using a 4% chloral hydrate solution intraperitoneally, followed by blood collection via orbital extraction. Blood samples were centrifuged at 3500 rpm for 20 min at 4°C, with serum stored at -80°C for analysis. Post-experiment, mice were euthanized and stomach, duodenum, and cecum tissues were extracted and fixed for histological examination.

### Staining of mice stomach and duodenal tissues

*H. pylori* presence in stomach tissues was detected using a modified Warthin-Starry (W-S) silver stain, effective in revealing *H. pylori* as black deposits in tissues (27). For structural and pathological assessments, tissues underwent H&E staining after fixing, dehydration, and paraffin embedding. Tissue sections of 4 μm were stained using Dong et al.’s (2022) methods to detail the tissues’ structural and pathological states (28).

### Detection of *H. pylori* colonization in mice stomach tissues

Gastric mucosa samples were incubated in a solution containing 1 g/mL urea and 850 µg/mL phenol red at 37°C for 30 min, observing color changes (29). Additionally, homogenized samples in PBS were diluted, plated on selective media, and cultured under microaerophilic conditions at 37°C for 2-5 days to identify *H. pylori* based on typical colony characteristics.

### Serological testing in mice

Post-experiment, anti-*H. pylori* IgG antibodies in mouse serum were detected using an ELISA (Yuanju Biotechnology Center, Shanghai, China). Serum samples, diluted 1:50, were added to *H. pylori* antigen-coated wells, incubated at 37°C for 30 min, washed thrice, and treated with HRP-labeled rabbit anti-mouse IgG for another 30 min. The reaction developed with Tetramethylbenzidine (TMB) was stopped using sulfuric acid and read at 450 nm. Serum was analyzed for gastric enzymes (α-amylase, pepsin, cellulase), inflammatory markers (Interleukin-17A (IL-17A), Interleukin-23 (IL-23), Interleukin-10 (IL-10), Interferon beta (IFN-β), Interferon gamma (IFN-γ), Forkhead Box P3 (FoxP3), endotoxin (ET), interferon–γ–inducible protein 10 (IP-10), transforming growth factor beta-1 (TGF-β1)), and activities of catalase (CAT), malondialdehyde (MDA), superoxide dismutase (SOD), and myeloperoxidase (MPO). All assays were performed according to the manufacturer’s instructions provided with each kit (BIOSCO Biotechnology Co., Ltd, Jiangsu, China).

### DNA extraction, 16S rRNA gene amplicon sequencing, and bioinformatics analysis of stomach and cecum microbiomes

We analyzed the microbial communities in stomach and cecum tissues via 16S rRNA gene sequencing. DNA was extracted, and the V3-V4 regions of the 16S rRNA gene were amplified using 341F and 805R primers. The PCR process involved an initial denaturation at 95°C, followed by cycles at 94°C for denaturation, 58°C for annealing, and 72°C for extension, concluding with a final extension at 72°C. Amplified products were indexed, pooled, and sequenced on an Illumina MiSeq platform in a paired-end 2x300 bp format. Raw data were refined using USEARCH to remove low-quality reads and chimeras. Amplicon sequence variants (ASVs) were clustered at 97% similarity with UPARSE (version 7.1) and classified via the RDP classifier (30). Alpha and Beta diversity were analyzed using QIIME, with significant differences between communities determined by linear discriminant analysis effect size (LEfSe), PICRUSt, and statistical analysis of metagenomic profiles (STAMP) analyses (31).

### Statistical analysis

All data were presented as mean ± standard deviation (SD). Statistical analyses were performed using R Studio software. The data were first tested for normal distribution using the Kolmogorov-Smirnov test. Multiple group comparisons were made using the non-parametric Kruskal-Wallis test, while differences between two groups were assessed using the Mann-Whitney U test, with a significance level set at p < 0.05. Microbial community analysis was conducted using QIIME software and principal coordinate analysis (PCoA) based on unweighted UniFrac distances (32). Community phylogenetics were investigated using PICRUSt and LEfSe analyses, while Spearman’s rank correlation test was used to explore correlations between bacterial genera in stomach and cecum tissues. All statistical analyses were performed in the R environment version 4.3.2 (33).

## Results

### *In vitro* test: CLSM validation of co-aggregation between Lp05 and SS1

**Figure 2:**
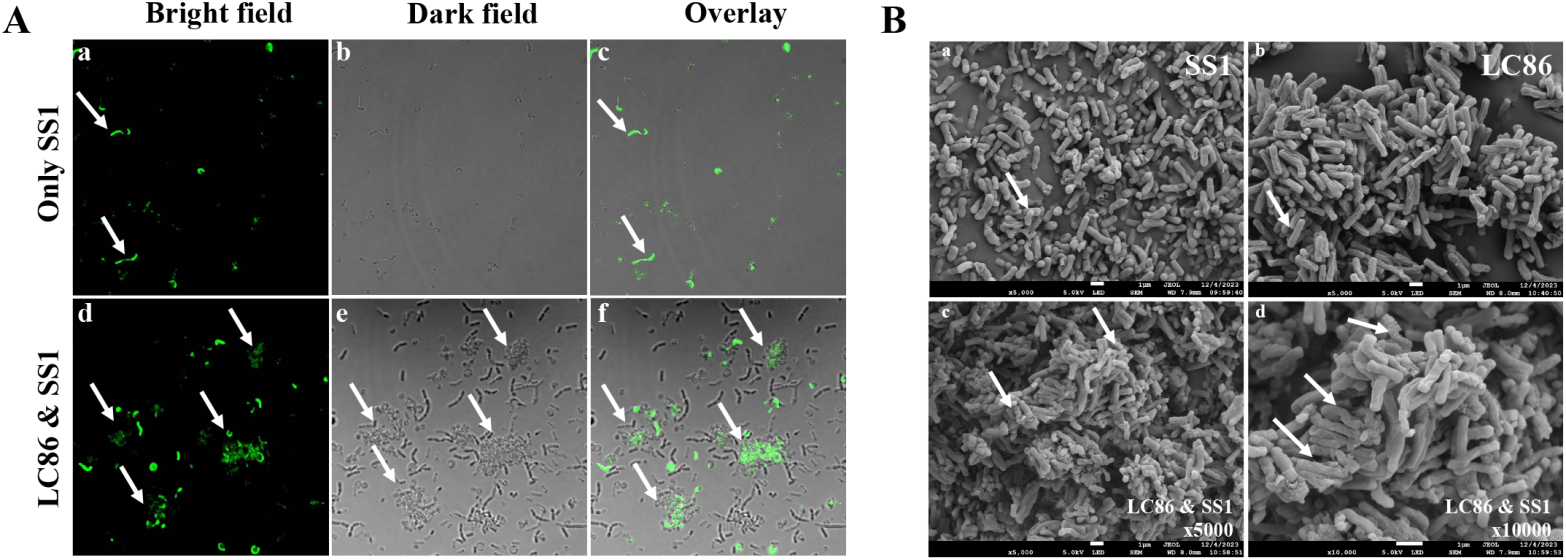
*In vitro* assessment of *Lactiplantibacillus plantarum* Lp05 inhibitory effect on *Helicobacter pylori* SS1. **(A)** Confocal laser scanning microscopy images after 2 h of co-aggregation between Lp05 and *H pylori* SS1; **(a–c)**: *H pylori* SS1 cultured alone; **(d–f)** Lp05 and *H pylori* SS1 co-cultured; **(a, d)** - bright field; **(b, e)** - dark field; **(c, f)** - overlay. **(B)** Scanning electron microscopy images of co-aggregates between Lp05 and *H pylori* SS1; **(a)** shows *H pylori* SS1; **(b)** shows Lp05; **(c, d)** show the co-aggregates **(c)** x5000, **(d)** x10000.

This study utilized CLSM to confirm the co-aggregation phenomenon between Lp05 and SS1. CFDA SE fluorescent staining was employed to label SS1, facilitating the identification of its presence within the co-aggregates. Observations under the microscope are presented in **Figure 2A (a-c)**, which shows the distribution of SS1 cultured alone under bright field, dark field, and overlay conditions, with bacteria evenly dispersed and no aggregates formed. In contrast, **Figure 2A (d-f)** displays the situation after 2 h of co-culturing Lp05 with SS1, where large co-aggregates (indicated by arrows) are visible in all three fields. These aggregates clearly demonstrate SS1 being encapsulated by Lp05, clustering together, which may facilitate its expulsion from the stomach, thus potentially reducing the gastric colonization of *H. pylori*.

### *In vitro* test: SEM characterization of the co-aggregation between Lp05 and SS1

The formation of co-aggregates between Lp05 and SS1 was also observed using SEM. After freeze-drying under vacuum, the aggregates were examined under a cold field emission scanning electron microscope to confirm the co-aggregation. **Figure 2B (a, b)** shows SS1 and Lp05 cultured separately, magnified 5000 times; SS1 appears as curved rods while Lp05 is depicted as short rods, with significant morphological differences between the two. **Figure 2B (c, d)** display the co-aggregates at magnifications of 5000 and 10000 times, respectively, with arrows pointing to multiple SS1 bodies bonded with Lp05, forming larger aggregates. Notably, the morphology of the Lp05 strain remains stable, whereas the co-cultured SS1 shows signs of edge dispersion, cellular deformation, collapse, and breakage, indicating significant impact on its cell structure. These observations confirm the effective formation of co-aggregates by Lp05 with *H. pylori*, reducing its gastric colonization.

### Effects of Lp05 on urease activity and *H. pylori*-IgG antibody levels in *H. pylori*-infected mice model

**Figure 3:**
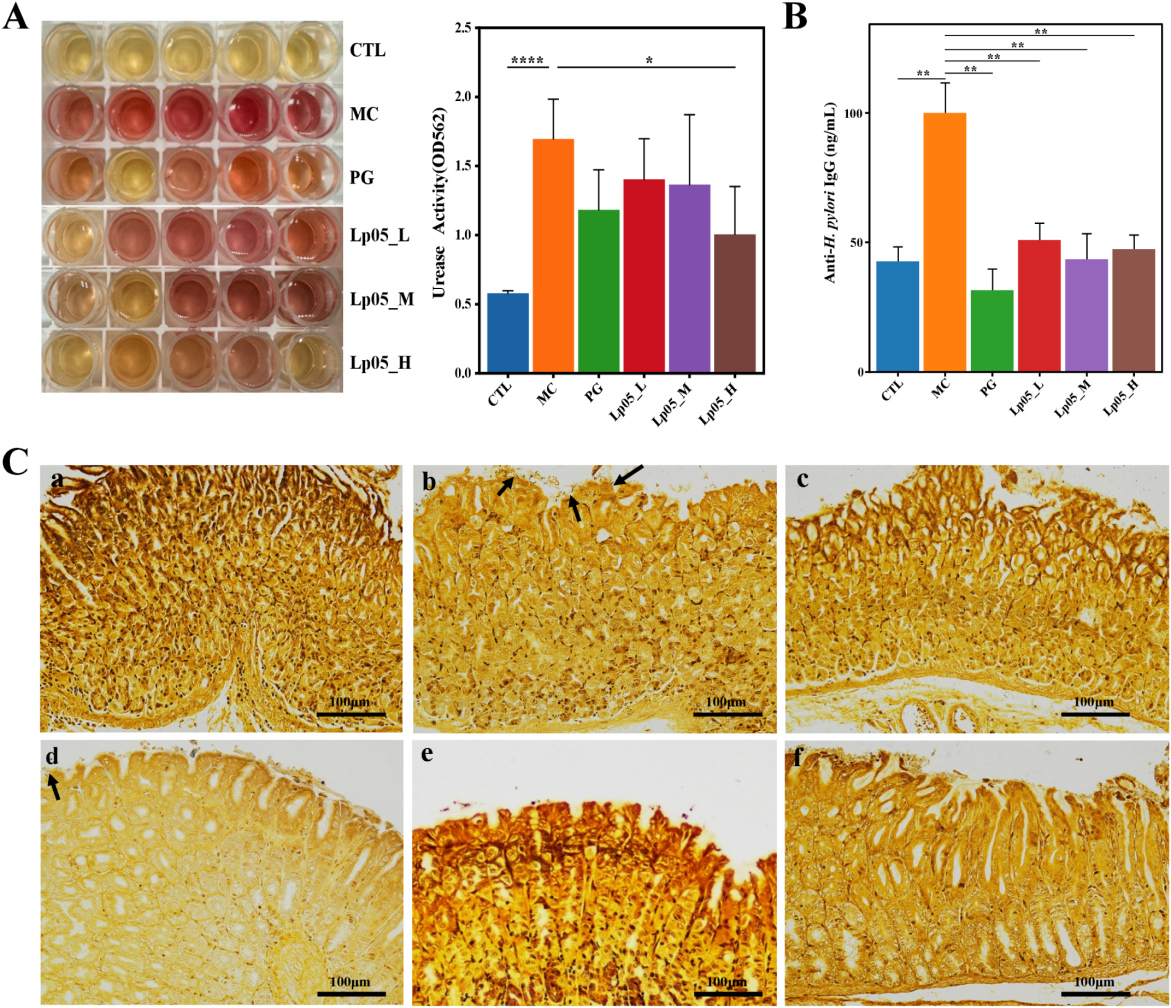
Effects of *Lactiplantibacillus plantarum* Lp05 on mice infected with *H pylori*. **(A)** Impact on gastric mucosal urease activity. **(B)** Levels of anti-*H. pylori* IgG antibodies in mouse serum. **(C)** Gastric tissue sections stained with Warthin-Starry silver staining; subpanels **a-f** correspond to CTL (control), MC (*H. pylori* infection model), PG (quadruple therapy group), Lp05_L (low dose, 2×10^7^ CFU), Lp05_M (medium dose, 2×10^8^ CFU), Lp05_H (high dose, 2×10^9^ CFU), respectively. *: p < 0.05, **: p < 0.01, ****: p < 0.0001.

The urease activity in gastric mucosa samples was assessed as shown in **Figure 3A**. Notably, the MC group exhibited significantly increased urease activity, leading to a rise in pH that changed the phenol red indicator from yellow to pink or red. Quantitatively, urease activity in the MC group was markedly higher, with an optical density (OD) measurement at 562 nm reaching 2.0. In comparison, groups treated with various doses of Lp05 and the PG group demonstrated lower urease activities, with minimal change in the color of the phenol red indicator or maintenance of its yellow color. Particularly, the Lp05_H group showed urease activities comparable to those of the PG group (OD_562nm_ = 0.5) and significantly lower than those in the MC group. This suggests that Lp05 can reduce gastric colonization by *H. pylori*, thereby lowering urease activity induced by *H. pylori* infection and alleviating tissue inflammation caused by *H. pylori* colonization and infection. These findings are substantiated by the OD measurements, indicating effective suppression of urease by Lp05_H treatment. Additionally, serum levels of *H. pylori*-IgG antibodies (as depicted in **Figure 3B**) were significantly lower (*p* < 0.01) in mice treated with Lp05 compared to the MC group, approaching levels seen in the CTL group and comparable to those in the PG group. This indicates that Lp05 can effectively reduce the systemic immune response to *H. pylori*.

### Impact of Lp05 intervention on gastric tissue structure in *H. pylori*-infected mice model

**Figure 3C** presents W-S silver-stained sections of mouse gastric tissue. Compared to the CTL group, the MC group displayed dark brown to black rod-shaped or slightly helical bacteria (indicated by arrows) on the mucosal layer, epithelial surface, and intercellular spaces. *H. pylori* predominantly colonized the gastric pits and gland lumina at the junction of the gastric body and antrum, generally appearing arc-shaped, S-shaped, and occasionally as short rods. After homogenizing gastric tissues and culturing on Brucella agar plates for three days, Gram staining of the colonies (**Supplementary Figure S1**) revealed typical red S-shaped, slightly helical, or curved rod-shaped morphology of *H. pylori*, confirming its Gram-negative nature and successful establishment of the *H. pylori* infection model in the MC group with observable colonization of the gastric mucosa. By contrast, in Lp05_L group, occasional *H. pylori* colonization was still visible. However, in Lp05_M and Lp05_H groups, the colonization of *H. pylori* was comparable to that in the CTL and PG groups, indicating that medium and high doses of Lp05 significantly inhibit the colonization of *H. pylori* on the gastric mucosa.

### Impact of Lp05 on gastric and duodenal tissue in *H. pylori*-infected mice model

**Figure 4:**
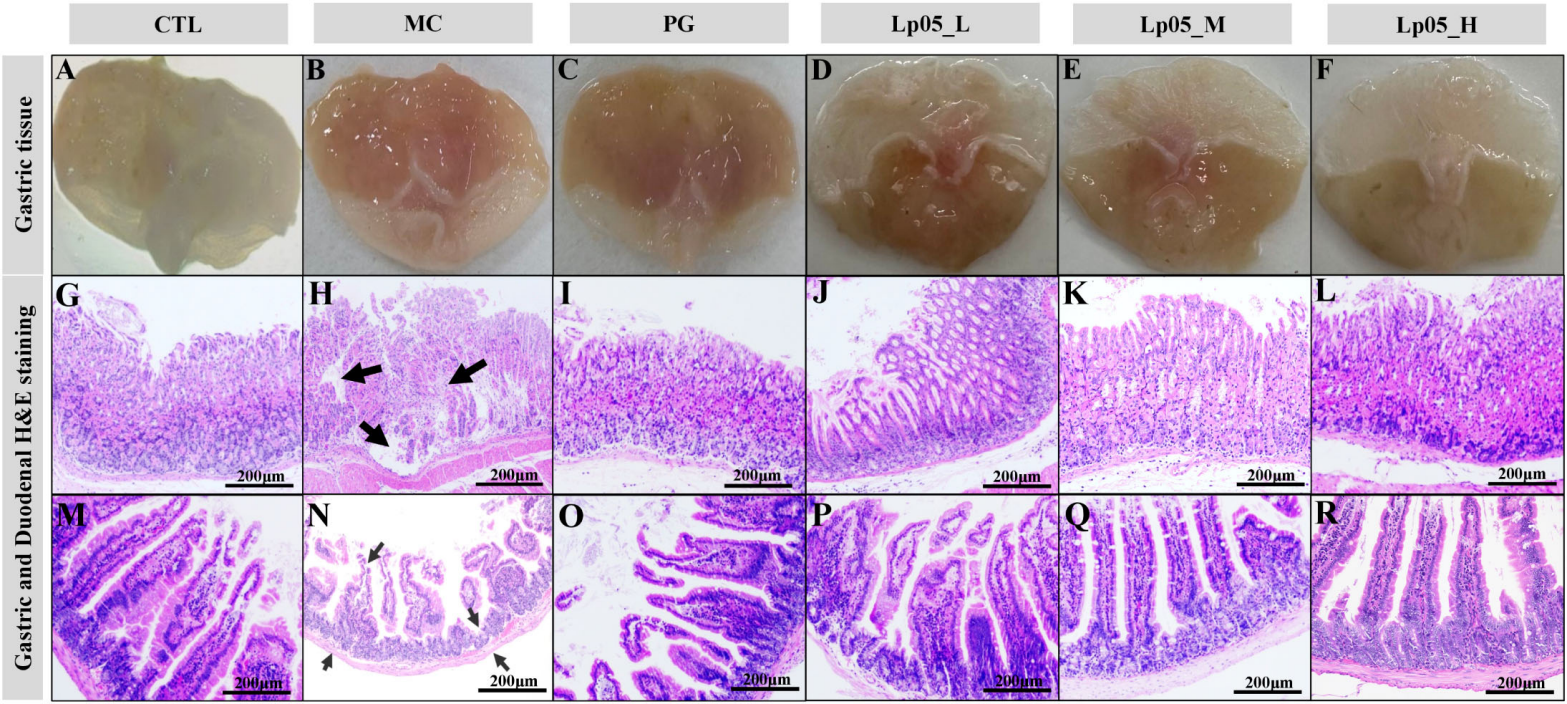
H&E staining of gastric and duodenal tissues in mice infected with *H. pylori* across *Lactiplantibacillus plantarum* Lp05 dosages. **(A–L)** Pathological images of gastric tissue HE stains; **(M–R)** Pathological images of duodenal tissue HE stains. Groups, CTL (control), MC (*H. pylori* infection model), PG (quadruple therapy group), Lp05_L (low-dose, 2×10^7^ CFU), Lp05_M (medium-dose, 2×10^8^ CFU), Lp05_H (high-dose, 2×10^9^ CFU).

HE staining revealed the effects of Lp05 on gastric and duodenal tissues in *H. pylori*-infected mice. **Figures 4A–L** show that in the CTL group, gastric structures were intact with well-defined gastric pits, orderly epithelial cells, and no inflammation. Conversely, the MC group exhibited *H. pylori*-induced pathologies such as epithelial necrosis, disorganized glands, and inflammation, with fewer parietal cells. The PG group had mild inflammation and minor ulcers. The Lp05_L group showed mild to moderate inflammation, whereas the Lp05_M and Lp05_H groups demonstrated notable tissue improvements, especially the Lp05_H group, where the gastric mucosa was clear, intact, and exhibited minimal inflammation, similar to the CTL group. For the duodenum, **Figures 4M–R** indicates regular mucosal structures in the CTL group, while the MC group showed extensive cellular necrosis and inflammation, disrupting the structure significantly. Despite treatment, the PG group had disordered epithelial cells. Post Lp05 treatment, especially in the Lp05_M and Lp05_H groups, the duodenal villi were intact with regular epithelial cell arrangements, suggesting that medium and high doses of Lp05 effectively alleviate *H. pylori*-induced duodenal damage.

### Effects of Lp05 on digestive enzymes in gastric tissue in *H. pylori*-infected mice model

**Figure 5:**
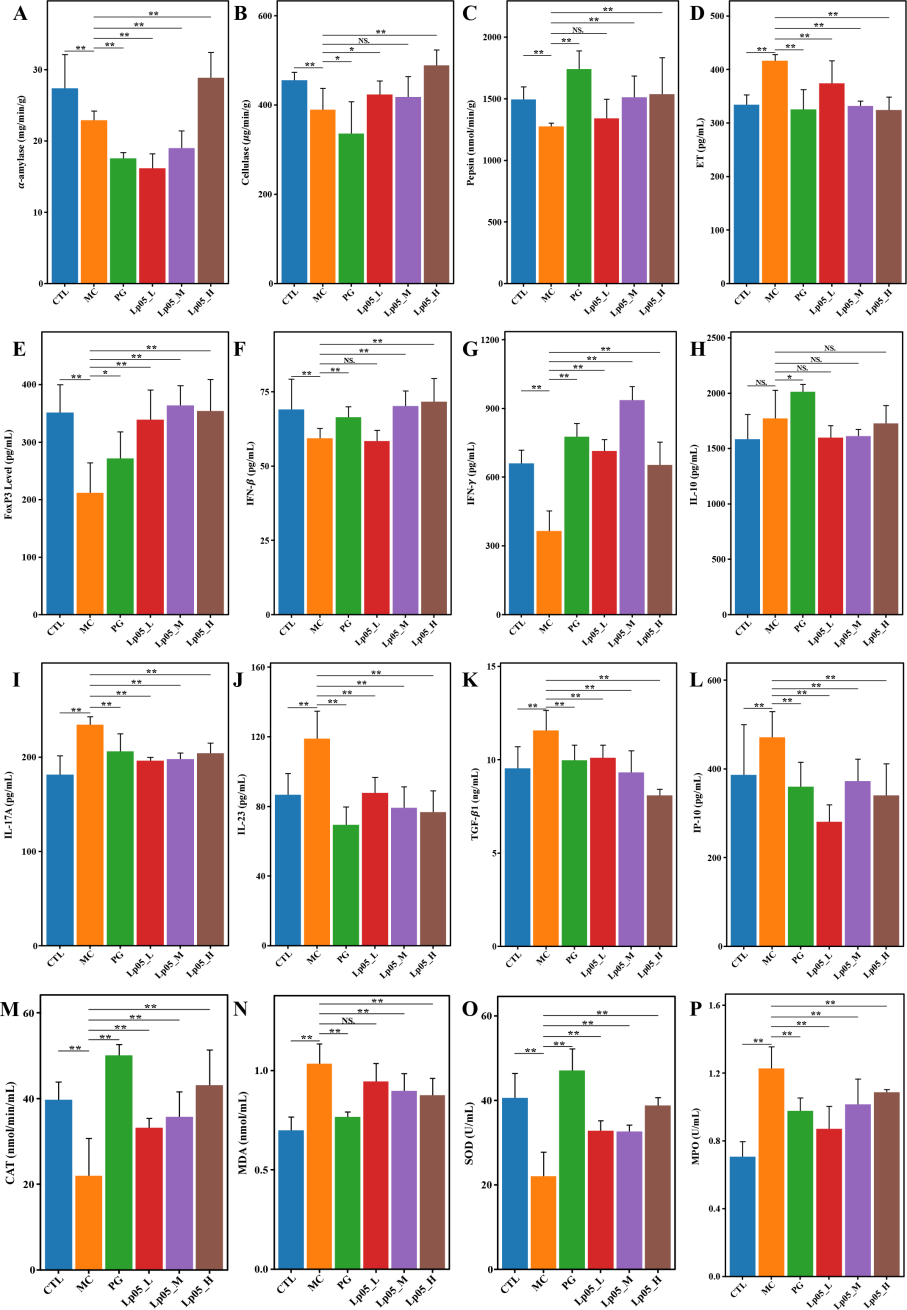
Effects of *Lactiplantibacillus plantarum* Lp05 on pepsin activity, immune response, and oxidative stress levels in mice infected with *Helicobacter pylori*. **(A–C)** Activities of gastric proteases: α-amylase, cellulase, and pepsin; **(D–L)** Levels of ET, FoxP3, IFN-*β*, IFN-*γ*, IL-10, IL-17A, IL-23, TGF- *β*1, IP-10; **(M–P)** Levels of CAT, MDA, SOD, and MPO. ** indicates *p* < 0.01, * indicates *p* < 0.05. NS indicates p > 0.05.

This test assessed the impact of Lp05 on digestive enzymes in the gastric tissue of mice infected with *H. pylori*, focusing on α-amylase, cellulase, and pepsin. Specifically, in the CTL group, the mean α-amylase activity was 27.5 mg/min/g. Post-infection in the MC group, activity decreased significantly to 17.2 mg/min/g (*p* < 0.01), indicating the detrimental effects of *H. pylori* infection. Following treatment with Lp05, α-amylase activity in the Lp05_H group almost completely recovered to 28 mg/min/g, slightly surpassing the CTL group. The baseline pepsin activity in the CTL group was 1501 nmol/min/g. After *H. pylori* infection (MC group), activity dropped to 1260 nmol/min/g (*p* < 0.01). Remarkably, pepsin activity increased to 1614.9 nmol/min/g in the Lp05_H group, significantly higher than the CTL group, which underscores the effectiveness of Lp05 in not only reversing the infection impact but also enhancing enzyme activity. For cellulase, the activity was 454.9 μg/min/g in the CTL group, reduced to 329.5 μg/min/g in the MC group (*p* < 0.01) due to *H. pylori* infection. Post-Lp05 treatment, activity in the Lp05_H group reached 481.6 μg/min/g, again exceeding the CTL, indicating significant recovery and enhancement of function. These results (**Figures 5A–C**) suggest that Lp05 intervention, particularly at higher doses, supports overall gastric function recovery by normalizing and enhancing digestive enzyme activities, which are otherwise compromised by *H. pylori* infection.

### Impact of Lp05 on serum inflammatory cytokines in *H. pylori*-infected mice model

As shown in **Figures 5D–L**, in the MC group, ET levels were measured at 420.1 pg/mL, elevated compared to the CTL group, which had levels of 331 pg/mL. The Lp05_H group effectively reduced ET to 332.24 pg/mL (*p* < 0.05), closely aligning with the CTL group. IL-17A was higher in MC at 236.15 pg/mL compared to 186.3 pg/mL in CTL, and was reduced to 204 pg/mL in Lp05_H (*p* < 0.05). IL-23 levels rose to 117.5 pg/mL in MC from 83.9 pg/mL in CTL, but were moderated to 75.70 pg/mL with Lp05 treatment (*p* < 0.05). IP-10 levels increased to 495.5 pg/mL in MC from 387.1 pg/mL in CTL, with a reduction to 348.7 pg/mL observed in Lp05_H (*p* < 0.05). TGF-β1 also showed an increase in MC to 11.5 pg/mL from 9.4 pg/mL in CTL (*p* < 0.05), and was reduced to 8.7 pg/mL in Lp05_H (*p* < 0.05), indicating a substantial reduction. Conversely, IFN-β and IFN-γ, which decreased to 58.9 pg/mL and 391 pg/mL in MC from CTL levels of 67.7 pg/mL and 646.5 pg/mL respectively, were restored in Lp05_H to 67.8 pg/mL and 672.2 pg/mL (both *p* < 0.05). Additionally, FoxP3 levels were reduced in MC to 206.76 pg/mL from 343 pg/mL in CTL and were restored in Lp05_H to 341.0 pg/mL (*p* < 0.05). The effects of Lp05 were similar to those of PG group, particularly in Lp05_H group, where the modulation of inflammatory cytokines was especially pronounced, indicating that Lp05 effectively suppresses the systemic inflammatory response caused by *H. pylori* infection.

### Impact of Lp05 on oxidative stress response in *H. pylori*-infected mice model

As depicted in **Figures 5M–P**, oxidative stress markers were quantitatively analyzed to evaluate the impact of *H. pylori* infection and subsequent treatments. In the CTL group, CAT activity was measured at 38.1 nmol/min/mL, and SOD activity at 40.4 U/mL. Following *H. pylori* infection, the MC group exhibited significant reductions in these antioxidant enzymes, with CAT decreasing to 21.3 nmol/min/mL and SOD to 23.3 U/mL (all *p* < 0.05). This decline coincided with increases in MDA and MPO levels, from 0.72 nmol/mL to 1.03 nmol/mL and from 0.75 U/mL to 1.24 U/mL respectively, indicating a marked increase in oxidative stress. Post-treatment analyses showed significant reversals in these oxidative stress markers across all intervention groups (all *p* < 0.05). After treatment with varying doses of Lp05, CAT levels were increased to 42.2 nmol/min/mL in the Lp05_H group, surpassing CTL levels. Similarly, SOD levels improved to 38.2 U/mL in Lp05_H, nearly reaching CTL values. MDA and MPO levels were significantly reduced to 0.86 nmol/mL and 1.09 U/mL, respectively, in the Lp05_H group. These results suggest that high-dose Lp05 possesses potent antioxidant capabilities, effectively alleviating the oxidative damage caused by *H. pylori* infection.

### Impact of Lp05 on gastric microbiome structure and function in *H. pylori*-infected mice model

**Figure 6:**
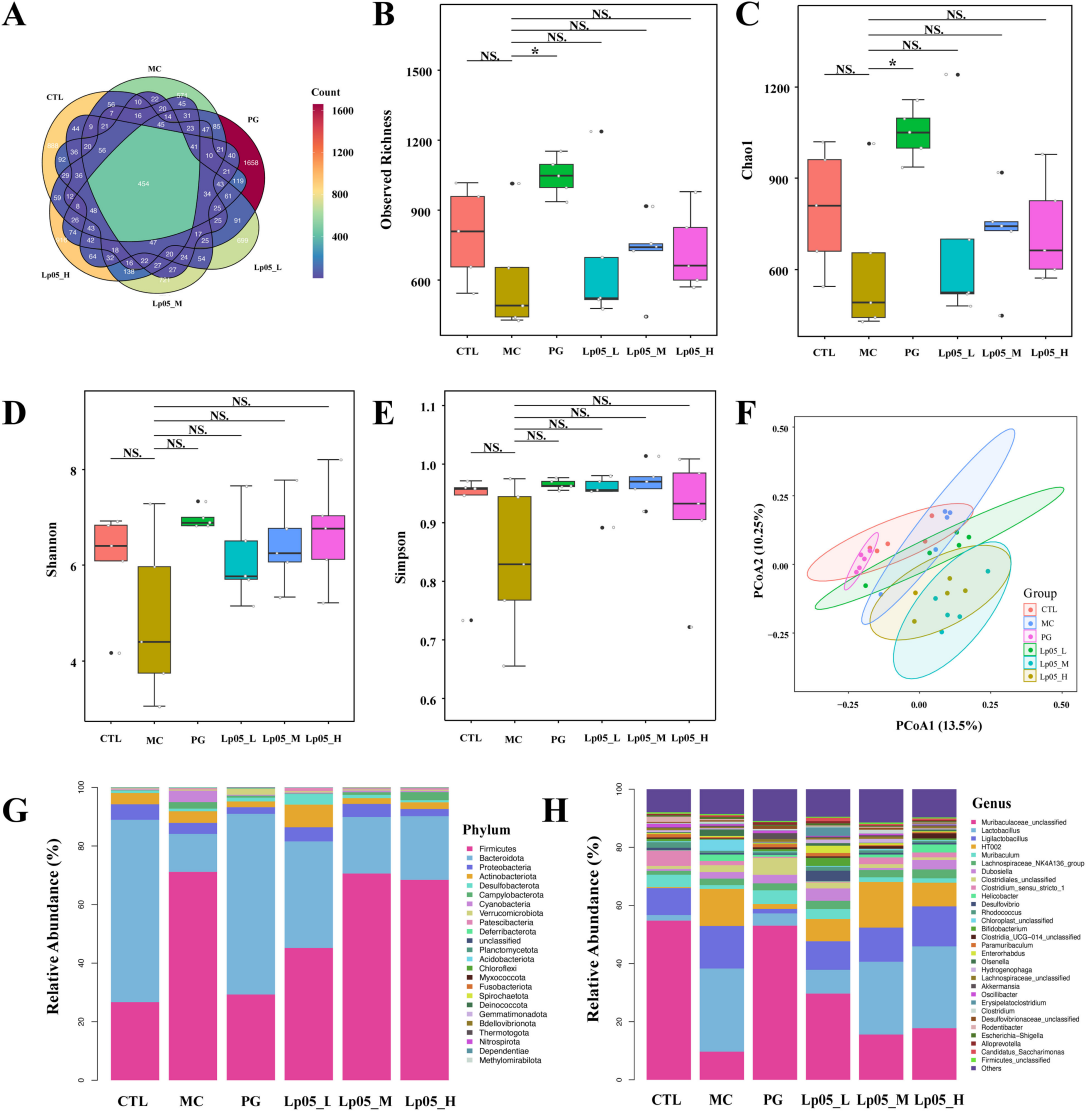
Impact of *Lactiplantibacillus plantarum* Lp05 doses on gastric microbial composition and structure. **(A)** Venn diagram analysis of the gastric microbiome sequencing; **(B–E)** Alpha diversity analysis of the gastric microbiome; **(F)** Beta diversity analysis of the gastric microbiome through PCoA; **(G, H)** Relative abundance of species at the phylum and genus levels in the gastric microbiome; CTL, control group; MC, *H pylori* infection model group; Lp05_L, low-dose group (2×10^7^ CFU); Lp05_M, medium-dose group (2×10^8^ CFU); Lp05_H, high-dose group (2×10^9^ CFU). NS indicates p > 0.05,* indicates p < 0.05.

**Figure 7:**
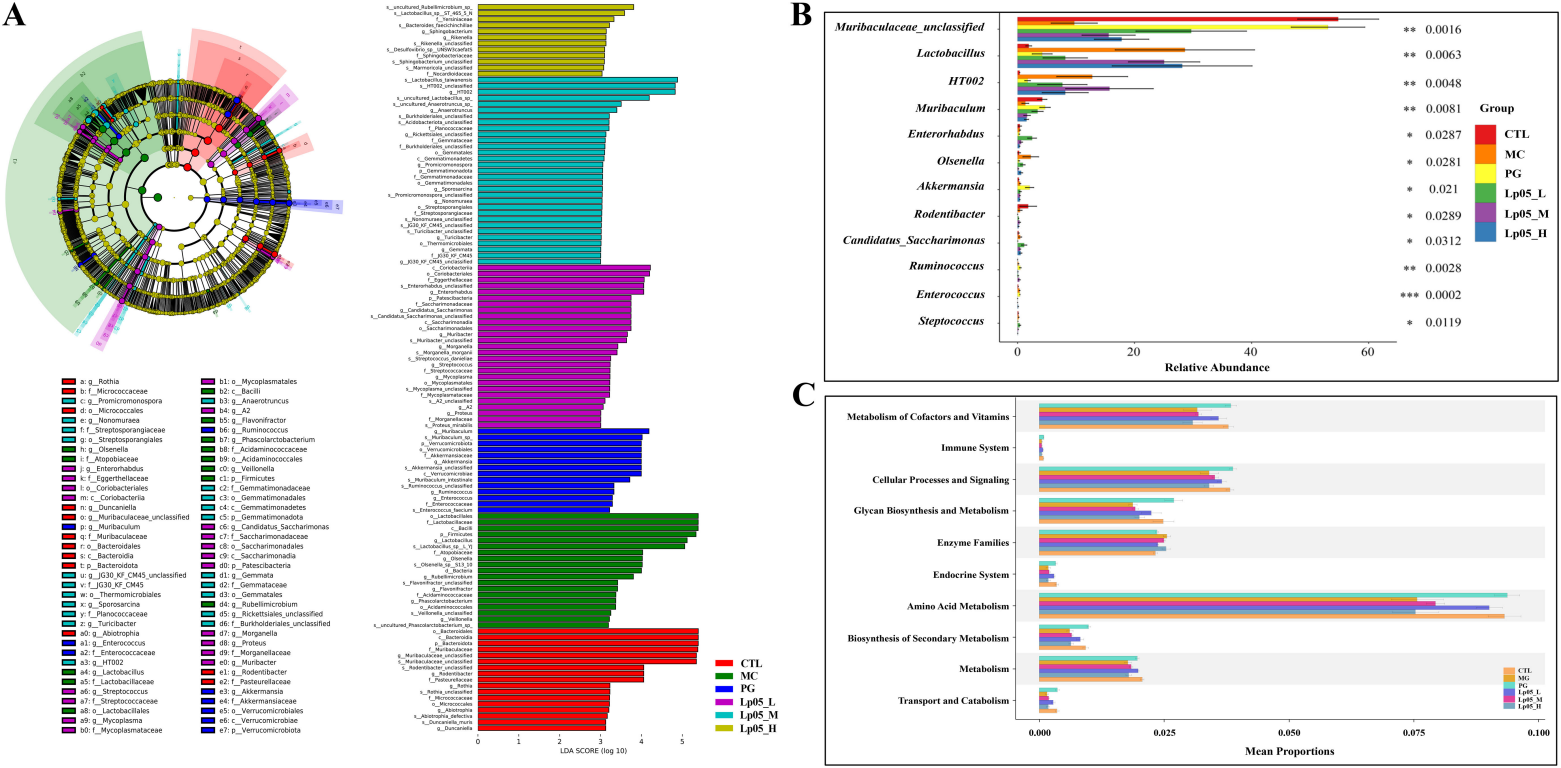
Effects of *Lactiplantibacillus plantarum* Lp05 doses on gastric microbial species abundance. **(A)** Phylogenetic cladogram and LefSe analysis LDA score histograms generated from metagenomic data; circle size is proportional to the abundance of taxonomic units. **(B)** STAMP analysis of differences at the genus level in the gastric microbiome. **(C)** PICRUSt analysis predicts the functions of gastric microbiome taxa at KEGG level 2.

The 16S rRNA gene sequencing assessed Lp05 effect on the gastric microbiome of H. pylori-infected mice. **Figure 6A** identifies 13,888 ASVs, with 420 common across groups. Unique ASVs were 2366 in the CTL group, 1815 in MC, 3046 in PG, and 2041, 2002, and 2198 in the low, medium, and high dose Lp05 groups respectively, indicating significant microbial diversity changes due to treatment. Observed richness, Chao1, Shannon, and Simpson indices (**Figures 6B–E**) evaluated microbial abundance, diversity, and evenness, showing a decrease in α-diversity in the MC group compared to CTL. Post-BQT and Lp05 interventions, richness and Chao1 indices increased, albeit not significantly versus the MC group. β-diversity analysis (**Figure 6F**) showed distinct microbial structures, with significant composition shifts in Lp05, MC, and PG groups compared to CTL. Dominant species and their abundances are detailed in **Figures 6G, H**. Firmicutes and Bacteroidota were predominant, with significant variations in relative abundance, for instance, 71.18% in MG versus 26.67% in CTL. Lp05 significantly adjusted these proportions, particularly in the low-dose group, aligning more closely with CTL. Dominant genera included *Muribaculaceae_unclassified*, *Lactobacillus*, and *Muribaculum*, with *Muribaculaceae_unclassified* showing increased abundance in PG and Lp05 groups versus MC, suggesting effective microbial modulation. LEfSe analysis (**Figure 7A**) highlighted marker species with significant differences across groups, showing beneficial genera like *Rikenella* were enhanced by Lp05, indicating its role in optimizing the gastric microbiome. STAMP analysis (**Figure 7B**) noted a significant recovery in *Muribaculaceae_unclassified* in Lp05-treated groups compared to MC, particularly in low doses. PICRUSt2 analysis (**Figure 7C**) predicted upregulated metabolic pathways such as amino acid metabolism and polysaccharide biosynthesis in Lp05 groups, essential for microbial community stability and nearing levels in the CTL group.

### Impact of Lp05 on the intestinal microbiome structure and function in *H. pylori*-infected mice model

**Figure 8:**
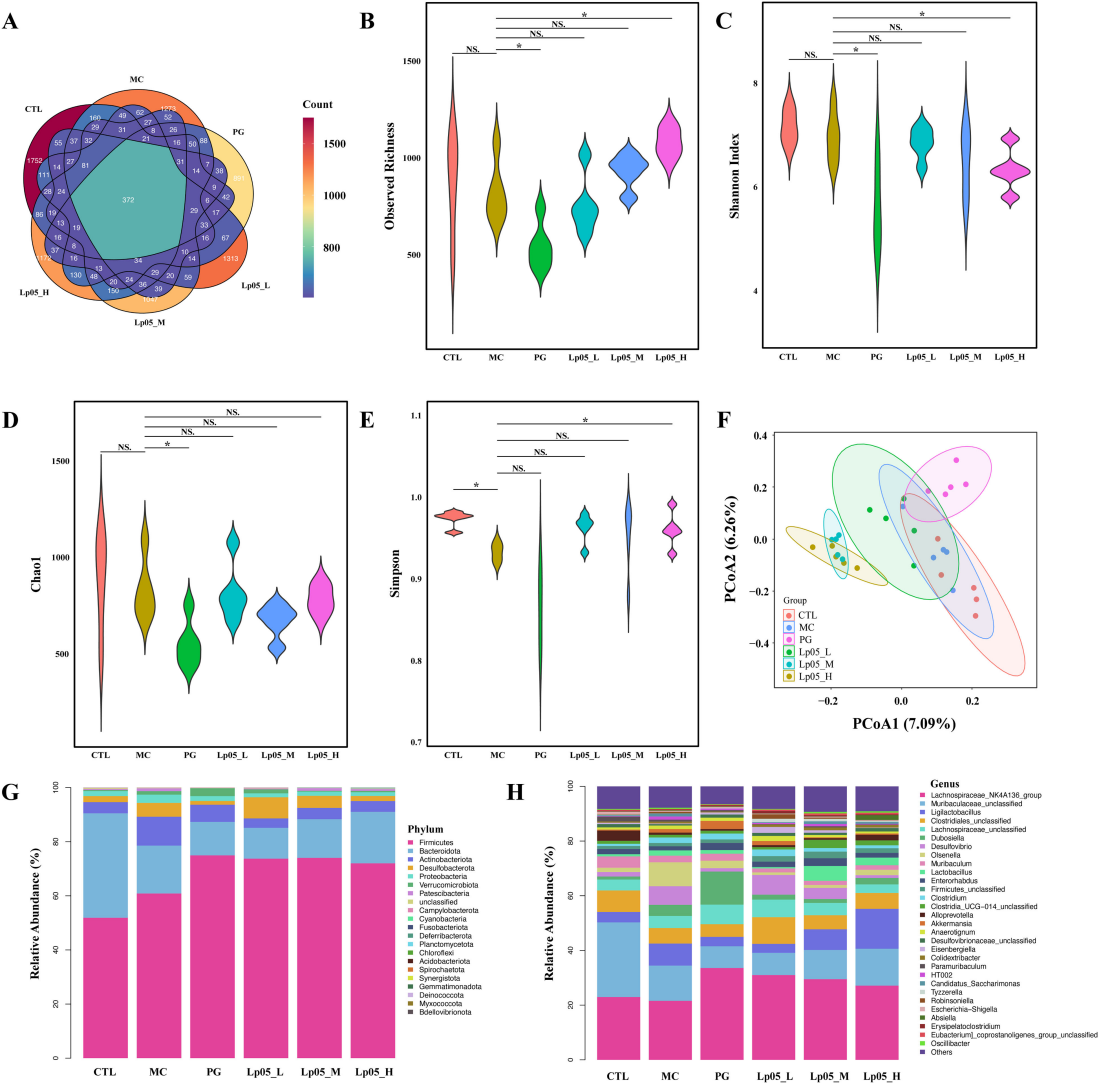
Impact of *Lactiplantibacillus plantarum* Lp05 doses on cecal microbial composition and structure. **(A)** Venn diagram analysis of intestinal microbiota sequencing; **(B–E)** Alpha diversity metrics of the intestinal microbiota; **(F)** Beta diversity of the intestinal microbiota assessed via PCoA analysis; **(G, H)** Relative abundance of species at the phylum and genus levels within the intestinal microbiota. Groups, CTL (control), MC (*H. pylori* infection model), Lp05_L (low dose, 2×10^7^ CFU), Lp05_M (medium dose, 2×10^8^ CFU), Lp05_H (high dose, 2×10^9^ CFU). NS indicates p > 0.05,* indicates p < 0.05.

**Figure 9:**
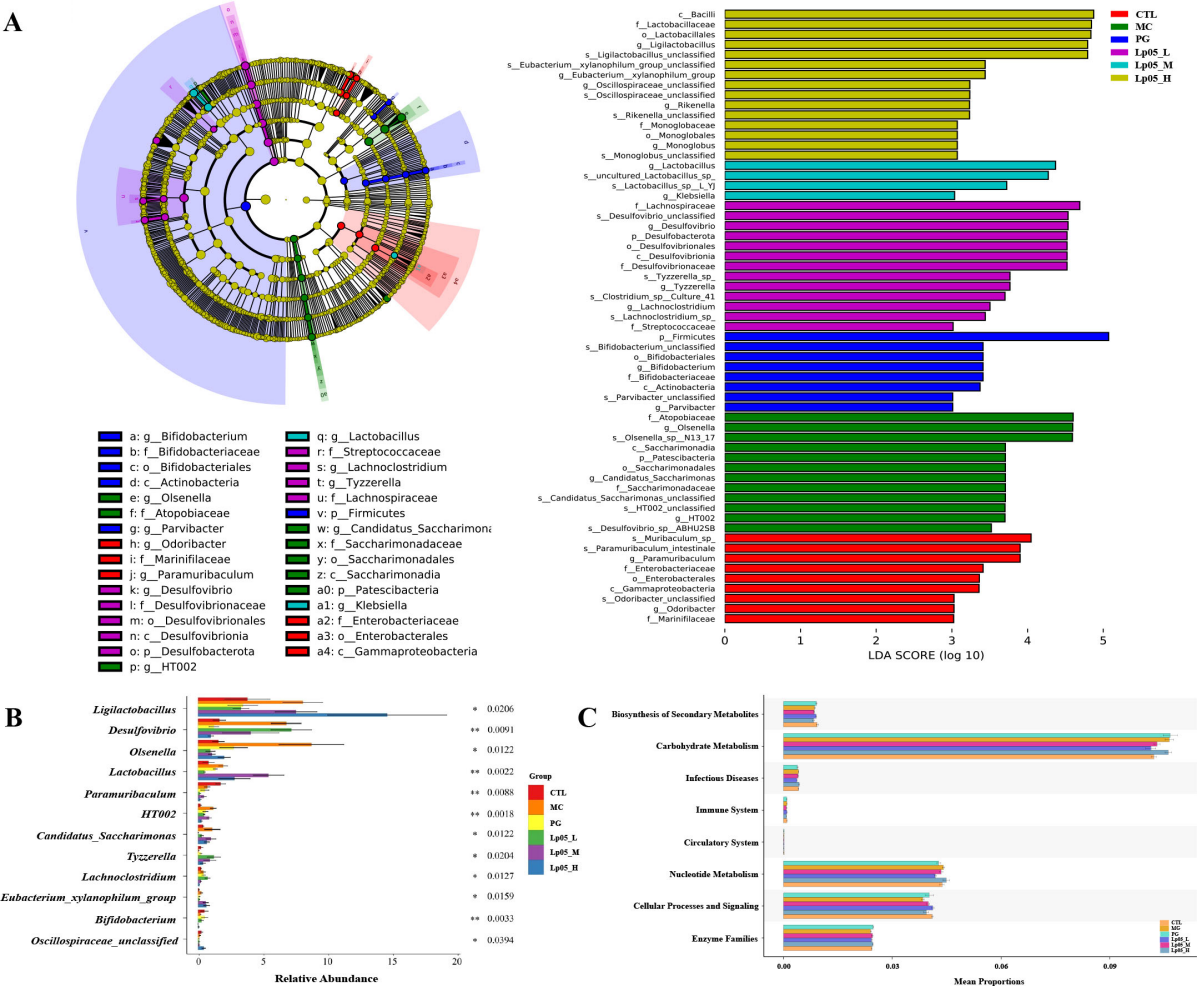
Effects of *Lactiplantibacillus plantarum* Lp05 doses on cecal microbiome diversity. **(A)** Cladogram from metagenomic data and LEfSe analysis showing taxa with significant differences, with circle sizes reflecting taxa abundance. **(B)** STAMP analysis identifies genus-level differences within the intestinal microbiome. **(C)** PICRUSt predicts functions of KEGG level 2 taxa in the intestinal microbiome. Groups, CTL (control), MC (*H. pylori* infection model), Lp05_L (low dose, 2×10^7^ CFU), Lp05_M (medium dose, 2×10^8^ CFU), Lp05_H (high dose, 2×10^9^ CFU).

Venn diagram analysis (**Figure 8A**) showed 14,342 ASVs, with 340 shared across all six groups. Unique ASVs were 2760 in CTL, 2518 in MC, 1627 in PG, and 2595, 1894, and 2268 in the Lp05 low, medium, and high-dose groups, respectively, indicating significant impacts on intestinal microbiome structure. Alpha diversity metrics, including observed richness, Chao1, Shannon, and Simpson indices (**Figures 8B–E**), demonstrated a decline in microbial richness and diversity in the MC group compared to CTL. Post-Lp05 treatment, particularly at high doses, significantly enhanced these indices (*p* < 0.05), suggesting Lp05’s effectiveness in restoring diversity lost due to *H. pylori* infection. PCoA analysis (**Figure 8F**) revealed significant microbial composition shifts in the Lp05, MC, and PG groups relative to CTL, indicating effective microbiome rebalancing. **Figures 8G, H** detailed dominant species at various taxonomic levels, with Firmicutes and Bacteroidota being prevalent. For instance, Firmicutes comprised 60.85% in MC versus 51.91% in CTL. Genus level analysis showed prominence of *Lachnospiraceae_NK4A136_group* and *Muribaculaceae_unclassified*, with Lp05 notably enhancing *Lachnospiraceae_NK4A136_group*, highlighting its potential for supporting intestinal health. LEfSe analysis (**Figure 9A**) identified significant biomarkers, with dominant species in CTL including *Muribaculum* and *Paramuribaculum*. MC showed enrichment in *Atopobiaceae* and *Olsenella*, while PG’s biomarkers were primarily Firmicutes and *Bifidobacterium*. Lp05’s intervention highlighted beneficial biomarkers such as Bacilli and *Ligilactobacillus*, suggesting a reduction in potential pathogens. STAMP analysis (**Figure 9B**) at the genus level confirmed significant abundance changes in *Ligilactobacillus*, *Bifidobacterium*, and *Olsenella*, indicating their roles in *H. pylori*-associated pathologies. PICRUSt2 analysis (**Figure 9C**) revealed significant enrichment in metabolic pathways, particularly carbohydrate and nucleotide metabolism, essential for cellular growth and repair. This suggests that Lp05 intervention regulates metabolic activities in the gastric microbiome, potentially reducing metabolic disorders and long-term health issues associated with *H. pylori* infection.

### Correlation analysis of gastric and intestinal microbiome in *H. pylori*-infected mice model

**Figure 10:**
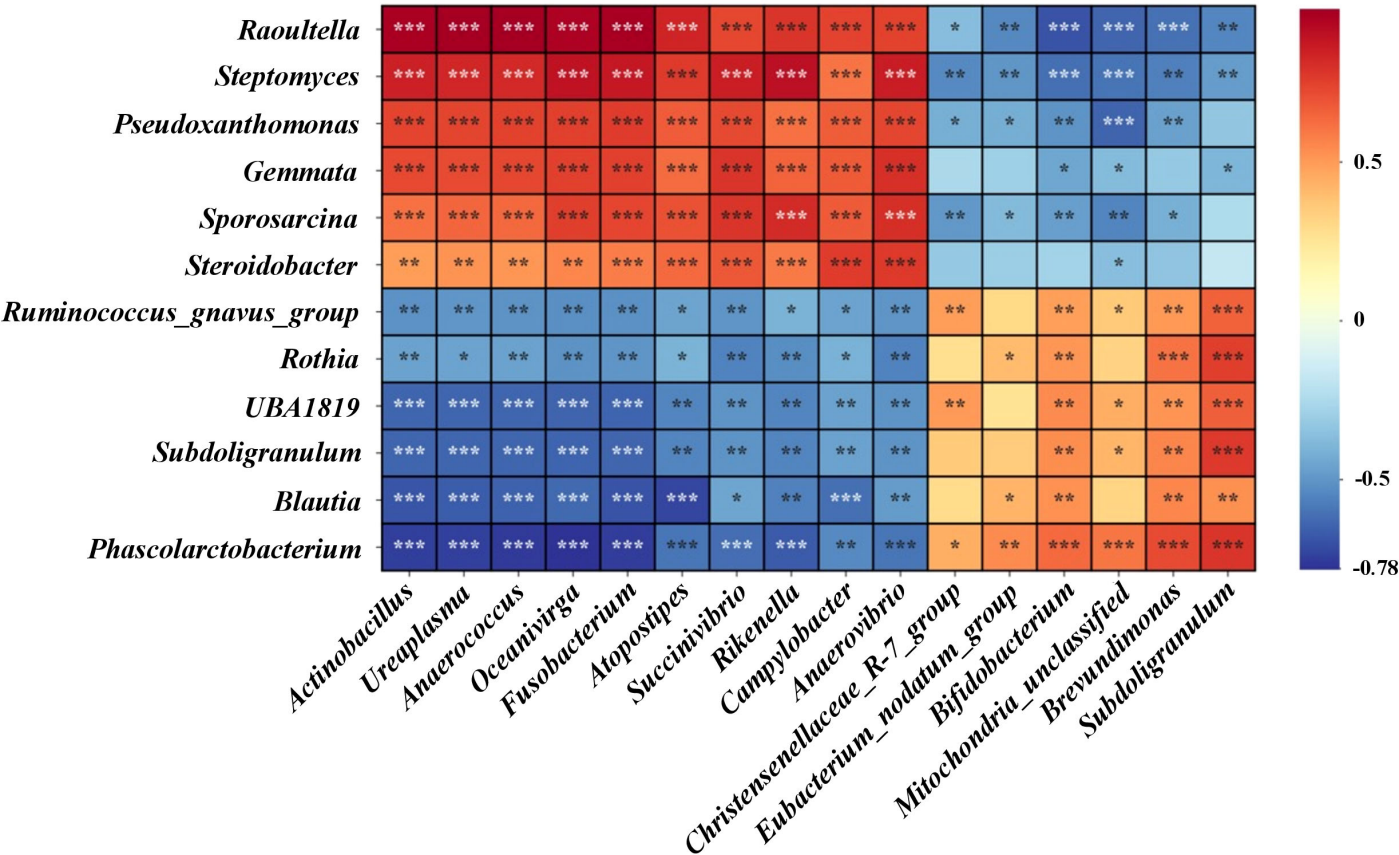
Presents the correlation analysis of differential microbial genera between gastric and intestinal microbiomes. Using Spearman's rank correlation test, the heatmap indicates significance levels with *** for *p* < 0.001, ** for *p* < 0.01, and * for *p* < 0.05.

Using Spearman’s correlation, we analyzed the relationship between the relative abundances of microbial genera in the gastric and intestinal microbiomes, as shown in **Figure 10**. In the gastric microbiome, genera such as *Raoultella*, *Streptomyce*s, *Pseudoxanthomonas*, *Gemmata*, *Sporosarcina*, and *Steroidobacter* were found to have significant positive correlations (*p* < 0.05) with intestinal genera including *Actinobacillus*, *Ureaplasma*, *Anaerococcus*, *Oceanivirga*, *Fusobacterium*, *Atopostipes*, *Succinivibrio*, *Rikenella*, *Campylobacter*, and *Anaerovibrio*. Conversely, these gastric genera showed negative correlations with *Christensenellaceae_R-7_group*, *Eubacterium_nodatum_group*, *Bifidobacterium*, *Mitochondria*, *Brevundimonas*, and *Subdoligranulum*. Additionally, gastric genera such as *Ruminococcus_gnavus_group*, *Rothia*, *UBA1819*, *Subdoligranulum*, *Blautia*, and *Phascolarctobacterium* demonstrated positive correlations with *Christensenellaceae_R-7_group*, *Eubacterium_nodatum_group*, *Bifidobacterium*, *Mitochondria*, *Brevundimonas*, and *Subdoligranulum*. However, these were significantly negatively correlated (*p* < 0.05) with the intestinal genera including *Actinobacillus*, *Ureaplasma*, *Anaerococcus*, *Oceanivirga*, *Fusobacterium*, *Atopostipes*, *Succinivibrio*, *Rikenella*, *Campylobacter*, and *Anaerovibrio*. This complex interplay of correlations between gastric and intestinal microbiomes indicates that Lp05 intervention not only affects specific genera but also influences the broader microbial ecosystem relationships within the host.

Using Spearman’s correlation, the study evaluated the relationships between gastric and intestinal microbial genera in *H. pylori*-infected mice, depicted in **Figure 10**. Gastric genera like *Raoultella*, *Streptomyces*, and *Pseudoxanthomonas* showed significant positive correlations (*p* < 0.05) with several intestinal genera such as *Actinobacillus* and *Ureaplasma*, indicating potential shared roles in gastrointestinal processes. Conversely, these gastric genera negatively correlated with *Christensenellaceae_R-7_group* and *Bifidobacterium*, suggesting differing physiological roles or adaptations to environmental conditions. Additionally, genera like *Ruminococcus_gnavus_group* displayed both positive and negative correlations with various intestinal genera, reflecting their complex interactions within the gastrointestinal tract. These findings highlight how Lp05 intervention impacts not only specific microbial genera but also the broader inter-genus relationships, affecting overall microbial ecosystem dynamics within the host.

## Discussion

This study demonstrates Lp05 potential as a therapeutic agent against *H. pylori* infection in mice, showcasing its antibacterial and immunomodulatory properties. Lp05 significantly reduces gastric mucosal urease activity, adjusts inflammatory cytokine levels, restores digestive enzyme functions, and alters gastric and intestinal microbiomes to combat *H. pylori*. These results not only underscore the effectiveness of probiotics in managing *H. pylori* infections but also highlight their capability to enhance gastrointestinal health. Lp05 may offer advantages over traditional treatments like quadruple therapy, potentially reducing antibiotic-associated gastric damage (34), which is crucial for long-term management.

Our findings are consistent with prior research on probiotics like *L. reuteri* and *L. johnsonii*, which have been shown to inhibit *H. pylori* growth and reduce gastric inflammation (18, 35). Specifically, Lp05’s effectiveness is demonstrated through *in vitro* studies where its co-aggregates with *H. pylori*, potentially disrupting its colonization by directly damaging its cellular structure or altering local pH levels (16, 36). *In vivo*, Lp05 significantly lowers urease activity and *H. pylori*-IgG levels in infected mice, suggesting reduced colonization and less gastric inflammation (17, 26, 37). Histopathological analysis shows Lp05 markedly improves tissue integrity in the stomach and duodenum, reducing inflammatory infiltration and maintaining epithelial structure, especially at higher doses. Unlike *L. reuteri* and *L. johnsonii*, Lp05 not only reduces urease activity and *H. pylori* colonization but also enhances the gastric environment and immune response, making it a comprehensive treatment option. These multifaceted effects of Lp05 highlight its potential as a holistic approach to managing *H. pylori* infection and promoting gastrointestinal health.

This study further examined the effects of Lp05 on the gastric microenvironment, focusing on antioxidant enzymes, digestive enzyme restoration, and immune modulation. Lp05 significantly restored the activity of essential digestive enzymes (α-amylase, cellulase, and pepsin) in the gastric tissues of *H. pylori*-infected mice. Typically, *H. pylori* infection impairs gastric function through mucosal damage and inflammation, which disrupt enzyme secretion and activity, and through the release of inflammatory mediators affecting enzyme function via complex signaling (38–40). Our findings show that Lp05 markedly enhances these enzymes’ activity, sometimes surpassing baseline levels. This suggests that Lp05 not only counters *H. pylori* colonization through direct antimicrobial effects but also facilitates recovery of gastric function, essential for combating *H. pylori*-induced dysfunction (6). Particularly at higher doses, Lp05’s restoration of enzyme activity underscores its multi-mechanistic approach, involving mucosal barrier repair, gastric secretion regulation, and reinstatement of normal gastric functions.

Additionally, Lp05 treatment significantly reduced the systemic inflammatory responses caused by *H. pylori* infection, which triggers both local gastric inflammation and systemic reactions, worsening the condition (41, 42). Following Lp05 intervention, inflammatory markers such as ET, IL-17A, IL-23, TGF-β1, and IP-10 were notably decreased, while regulatory markers like FoxP3, IFN-β, and IFN-γ were increased, indicating Lp05’s role in both reducing *H. pylori* colonization and modulating the host’s immune response. The high-dose group showed particularly strong improvements in inflammatory markers, underscoring Lp05’s effectiveness in immune modulation. Besides, oxidative stress, a key pathological response induced by *H. pylori*, involves reduced activity of antioxidant enzymes like CAT and SOD (43, 44), and increased oxidative markers like MDA and MPO (45, 46). Oxidative stress not only causes further damage to gastric tissues but may also trigger a range of downstream pathological responses, including cellular apoptosis and mutations (44, 47). Lp05 intervention significantly enhanced antioxidant enzyme activity and reduced oxidative stress markers, especially at higher doses, demonstrating its potential to mitigate oxidative stress and protect gastric tissues from *H. pylori*-induced damage. This suggests that probiotics could play a role in managing oxidative stress linked to infections.

We conducted further analyses of the gastric and cecal microbiota to explore how Lp05 mitigates *H. pylori* infection. The MC group displayed a marked decrease in alpha diversity, showing the pathogen’s impact on the host’s microbiome. Lp05 intervention significantly increased the observed richness and Chao1 indices in the gastric microbiome, suggesting it helps restore microbial diversity crucial for maintaining a healthy gastric environment and reducing *H. pylori* recurrence (48). Lp05 particularly raised the abundance of Firmicutes, known for their anti-inflammatory and protective functions (49), which may explain its effects on reducing inflammation. The recovery of Bacteroidota could relate to positive effects on intestinal barrier functions, aligning with improvements in intestinal structures (48, 50). Lp05 also significantly reduced urease activity, a crucial factor for *H. pylori*’s survival in acidic conditions (48). This suggests that Lp05’s modification of Firmicutes and Bacteroidota levels may alter gastric pH and microbial interactions, indirectly inhibiting urease activity (51, 52). LEfSe analysis showed that Lp05 enhanced bacterial taxa with anti-inflammatory and immunoregulatory functions like *Rikenella* (53, 54), correlating with decreases in inflammatory cytokines such as IL-10 and TGF-β1, indicating enhanced immune modulation. PICRUSt2 analysis revealed that Lp05 enriched pathways related to amino acid metabolism and polysaccharide biosynthesis, important for antioxidant defenses (55, 56). This corresponds with boosts in antioxidant enzyme activities such as CAT and SOD, suggesting that Lp05 promotes host antioxidant capabilities by modulating microbial communities and metabolic pathways, thus alleviating oxidative damage from *H. pylori* infection.

*H. pylori* infections disrupt gastric and intestinal microbial balance, evident from significant changes in biomarkers and microbial communities. Lp05 intervention notably restores diversity and richness in the intestinal microbiota, suggesting its role in rebalancing the microbial ecosystem to counteract *H. pylori* adverse effects. The observed enhancement in digestive enzyme activities, such as α-amylase, cellulase, and pepsin under Lp05 treatment, may directly relate to improved microbial health, as certain gut microbes influence the expression and activity of host enzymes, enhancing metabolic pathways and nutrient absorption (57, 58). LEfSe analysis revealed that Lp05 significantly alters microbial abundance related to health, particularly increasing the proportions of *Lachnospiraceae_NK4A136_group* and *Muribaculaceae_unclassified*, which could directly correlate with decreased levels of inflammatory factors like IL-17A, IL-23, and TGF-β1 (59). This increase in beneficial microbes might directly impact the host’s immune modulation by producing anti-inflammatory metabolites such as butyrate, thereby reducing inflammation (60). Additionally, Lp05 intervention reduces the abundance of *Olsenella*, reflecting its potential anti-inflammatory and gut health-promoting capabilities (61, 62). PICRUSt2 analysis of predicted functional changes shows that Lp05 influences microbial metabolic pathways, particularly enhancing carbohydrate and nucleotide metabolism, essential for cell growth and repair (63–65). This suggests that Lp05 not only affects the composition of the microbial community but also potentially improves metabolic health, which could help alleviate metabolic disturbances caused by *H. pylori* infections, reducing the risk of long-term health issues such as gastric cancer. Lp05’s intervention shows significant effects in reducing inflammation induced by *H. pylori*, adjusting the abundance of inflammation-related microbes and increasing anti-inflammatory microbial groups, which may help modulate the host’s immune response and reduce inflammation.

Further analysis of the interactions between gastric and intestinal microbiomes shows significant correlations between certain genera, such as *Raoultella* and *Streptomyces* in the stomach, and *Actinobacillus* and *Fusobacterium* in the intestines. These positive correlations suggest joint participation in gastrointestinal processes and emphasize the microbial communities’ synergistic role in overall health and stress response. Conversely, negative correlations like that between the *Ruminococcus_gnavus_*group in the stomach and *Actinobacillus* in the intestines highlight their differing physiological roles or unique environmental adaptations. For example, the *Ruminococcus_gnavus_*group may support mucosal barrier integrity and anti-inflammatory functions in the stomach, whereas it may be linked to inflammation or pathogen defense in the intestines (66). This analysis underlines the importance of microbial communities in systemic health management, influencing nutrient absorption, immune regulation, and pathogen defense. Notably, the negative correlation of *Bifidobacterium* suggests its supportive role in intestinal health might decrease in the stomach due to the stomach’s acidic environment (67). These insights reveal the intricate interactions between gastric and intestinal microbiomes and highlight the importance of considering the systemic impact of microbial communities in managing gastrointestinal diseases, suggesting a holistic approach could lead to better microbial health and host disease management strategies.

Overall, this study has unveiled the potential of Lp05 in strategies against *H. pylori*, demonstrating that its intervention not only influences the microbial communities in the stomach and intestines but also exerts positive effects on host physiological functions such as digestive enzyme activities and immune responses. However, the study’s limitations include the use of a specific animal model that may not fully reflect the complex physiological and microbiotic environments of humans, and the inability to completely explore the causal relationships between changes in microbial communities and host pathology. These factors underscore the necessity for future research to further validate these findings under diverse host models and broader environmental conditions.

## Conclusion

This study has confirmed the efficacy of Lp05 in treating mice infected with *H. pylori*, illustrating its ability to modulate the gastrointestinal microbiota, alleviate inflammation and oxidative stress, and optimize host health. These results highlight the potential of Lp05 as a probiotic and provide a scientific basis for further research into its clinical applications. Future studies should delve deeper into its specific mechanisms of action to expand its use in the prevention and treatment of gastrointestinal diseases.

## Data Availability

The datasets presented in this study can be found in online repositories. The names of the repository/repositories and accession number(s) can be found below: https://www.ncbi.nlm.nih.gov/, PRJNA1138072 https://www.ncbi.nlm.nih.gov/, PRJNA1138073.
