# Supplementary figures and images for: Enhancement of host defense against Helicobacter pylori infection through modulation of the gastrointestinal microenvironment by Lactiplantibacillus plantarum Lp05
Source: Front Immunol. 2025 Jan 17;15:1469885. doi: 10.3389/fimmu.2024.1469885 (PMC11782045; doi:10.3389/fimmu.2024.1469885)

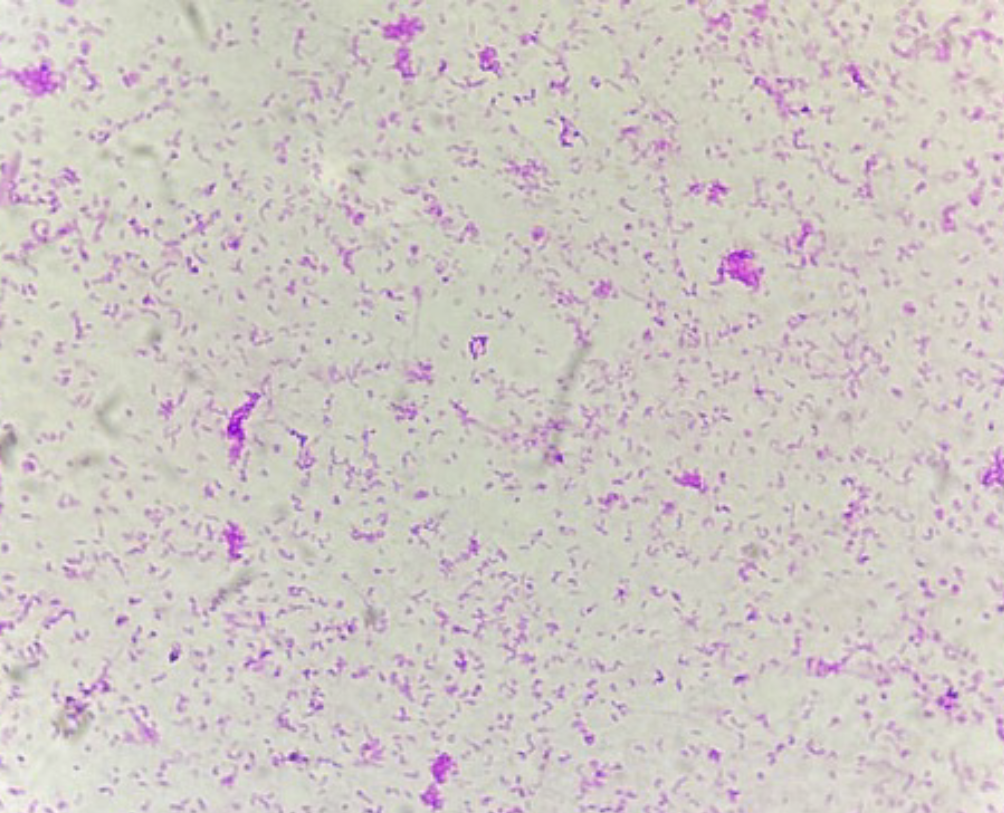

Supplement: Supplementary file 1 [file Image1.tif]
